# Milk consumers in Pakistan and Italy: a comparative study on the effects of geographical affiliation, socio-demographic characteristics and consumption patterns on knowledge, attitude and perception of antimicrobial resistance

**DOI:** 10.1186/s12889-024-21002-w

**Published:** 2024-12-18

**Authors:** Talal Hassan, Valentina Maria Merlino, Paola Badino, Rosangela Odore, Muhammad Qamer Shahid, Alberto Amerio, Manuela Renna

**Affiliations:** 1 Department of Veterinary Sciences, University of Turin, Grugliasco, TO Italy; 2 Department of Agricultural, Forest and Food Sciences, University of Turin, Grugliasco, TO Italy; 3 Department of Livestock Management, University of Veterinary and Animal Sciences, Lahore, Pakistan

**Keywords:** Antimicrobial resistance, Attitude, Consumer perception, Dairy sector, Knowledge, Low-to-middle income country, High-income country, Cluster analysis

## Abstract

Antimicrobials are extensively used in livestock to treat common diseases, including mastitis and other bacterial infections, with around 80% of food-producing animals receiving such treatments. Despite a ban in the EU since 2006, antibiotics are still used globally for growth promotion, exacerbating antimicrobial resistance (AMR). From the consumers’ point of view, the potential exposure to resistant zoonotic bacteria in animal-derived food products may represent a threat to public health. However, consumers’ knowledge and attitude vary across socio-demographic characteristics and geographical location. This study aims to evaluate the knowledge, attitudes, and perceptions (KAP) of milk consumers regarding AMR in Pakistan (as a representative of low-to-middle income countries (LMICs)), compared to Italy (as a representative of high-income countries (HICs)).

Data were collected via surveys from 401 Pakistani and 550 Italian milk consumers, focusing on socio-demographics, milk purchasing habits, and awareness of antibiotic residues and AMR. The study identified three principal components (PCs) influencing milk consumption: milk quality attributes and tradition, family-dependence, and eating style-dependence. A cluster analysis categorized consumers into four groups: "Attentive to milk quality attributes," "Loyal to milk," "Undecided consumer," and "Milk is essential in my food pattern."

Italian consumers predominantly belonged to the "Attentive to milk quality attributes" group, valuing safety and health benefits, and were the most informed about AMR. Pakistani consumers, primarily belonging to the "Loyal to milk" group, exhibited high purchasing frequency influenced by family size but showed limited awareness about AMR risks. The "Undecided consumer" group, a mix of Italian and Pakistani individuals, lacked strong preferences and displayed moderate awareness of AMR, influenced by habitual consumption patterns.

This research underscores the need for targeted educational campaigns to raise awareness about AMR and antibiotic residues in milk, particularly in LMICs. Survey findings can help to identify targeted action plans and boost policy development on AMR. Enhancing consumer knowledge through the One-Health approach could mitigate AMR risks and promote informed decision-making in milk consumption across different socio-economic contexts.

**Supplementary Information:**

The online version contains supplementary material available at 10.1186/s12889-024-21002-w.

## Background

Antimicrobials are mainly administered in livestock to treat common diseases, including mastitis, gastrointestinal and respiratory bacterial infections. According to recent surveys, about 80% of food-producing livestock undergo pharmacological treatments, including antibiotics. Moreover, although banned for growth promotion purposes in the EU since 2006, antibiotics are still improperly used in many countries worldwide to improve livestock productivity [1].

Antibiotic sales data are considered the most reliable indicator to estimate total antibiotic use in livestock. However, from a global perspective, the absence of harmonized surveillance systems and standardized collection methods pose an intrinsic limit to this approach [2]. Whereas the European Surveillance of Veterinary Antimicrobials Consumption (ESVAC) project has regularly provided data on the volume of sales of veterinary antimicrobial medicinal products in the European Union since 2009, in other low- and middle-income (LMIC) countries similar initiatives have yet to be implemented [3]. According to the latter authors, the global antimicrobial consumption in chickens, cattle and pigs is expected to increase by 11.5% over the next six years, with Asia being the largest consumer of antibiotics destined to farmed animals. Differences among countries can be attributed to local antimicrobial stewardship policies to mitigate the risk of antimicrobial resistance (AMR). It is in fact well established that a key driver for the emergence of AMR in pathogen and commensal bacteria is the use and/or the misuse of antimicrobials [4]. This, in turn, implies a risk for both animal and human health as resistance genes and bacteria can be transferred among animals and animal products via the food chain [5]. The potential exposure of consumers to resistant bacteria present in some food of animal origin, such as raw meat, milk and dairy products, is a matter of concern [6]. On the other hand, it has been postulated that chronic exposure of gut microbiota to antibiotic residues contained in food is a possible risk factor for AMR development [7]. In both cases, the risk for consumers mainly depends on food preparation (i.e., cooking method) and consumption habits. While researchers are aware of risks associated with the presence of antibiotic residues in food, public perception is not univocal. Consumers’ knowledge and attitude may vary across socio-demographic characteristics and geographical location [8]. Assessing and raising community awareness is a fundamental part of the One-Health approach to reduce inappropriate use of drugs and the risk of AMR development.

Due to the content of many essential nutrients, milk can be considered the most complete and fundamental food playing a key role in the diet of over 6 billion people worldwide [9]. Bovine mastitis is one of the most common diseases impacting on both milk yield and quality and, despite implementation of preventive control measures (e.g., pre- and post-milking teat disinfection), treatment mainly relies on the intramammary administration of antibiotics [10]. The use of veterinary drugs according to label prescriptions is critical to avoid drug residue concentrations in target tissues exceeding the maximum permitted levels (maximum residue levels—MRLs) [11]. Nevertheless, the problem of antibiotic residues remains topical in low-to-middle income countries due to the lack of effective surveillance systems and limited awareness of farmers and consumers about the possible threat for human health related to the presence of antimicrobials in milk [12]. According to a survey conducted in Pakistan on a total of 1,000 milk samples, about 10% of them showed concentrations of penicillin and oxytetracycline beyond the MRL fixed by the EU (4 µg/kg and 100 µg/kg, respectively) [13].

Although numerous studies have been conducted to assess consumers' knowledge, attitude, and perception (KAP) regarding antibiotic residues and AMR risks in the food chain, to our knowledge there is a lack of comparative studies between low-to-middle and high-income countries (LMIC and HIC, respectively) focusing on milk consumers. Therefore, the aim of the present study was to assess the effect of consumers’ geographical affiliation (LMIC – Pakistan *vs* HIC—Italy), socio-demographic characteristics, as well as purchasing and consumption habits of milk, on their KAP on AMR phenomenon.

## Methods

### Study area

Italy and Pakistan were selected for this study due to their contrasting socio-economic contexts, regulatory environments, and cultural practices related to food consumption. Italy, a developed country with stringent food safety regulations and a Mediterranean dietary culture, was chosen to represent a context where consumer behavior is influenced by both regulatory frameworks and cultural practices. Pakistan, a developing country with diverse dietary habits and less uniformly enforced food safety regulations, provides a contrasting environment where economic factors and cultural norms play a significant role in consumer behavior. These differences were deemed essential for exploring how varying contexts impact consumer attitudes towards milk consumption and food safety.

**Fig. 1:**
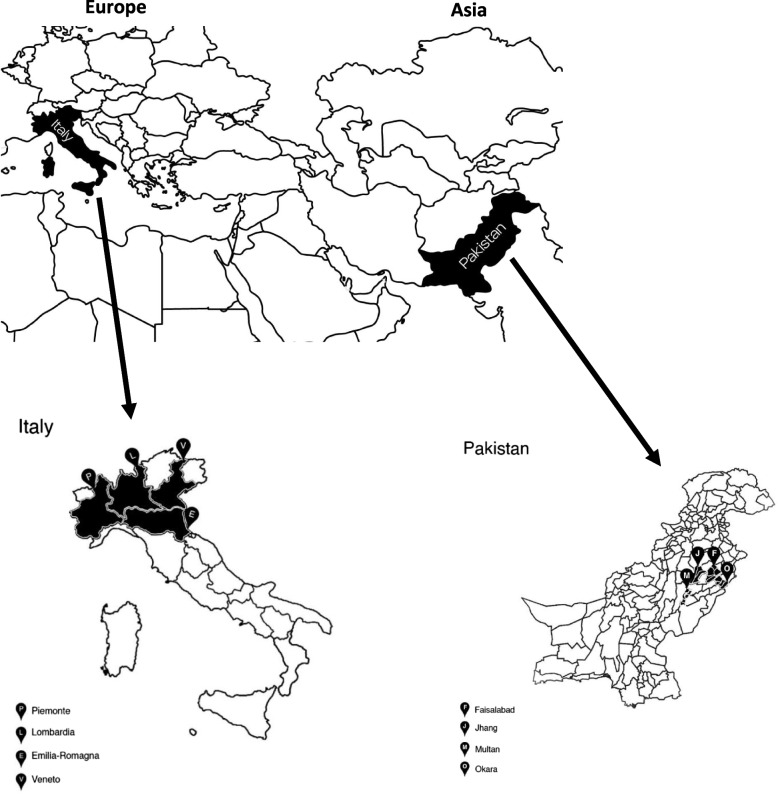
Geographical regions under study in Italy (Europe) and Pakistan (Asia)

The study area for each country was delimited to the main four regions where the largest national production of drinking milk is concentrated (Fig. 1). In Pakistan, data collection was concentrated in four districts (Faisalabad, Jhang, Multan, and Okara) belonging to the region of Punjab, where the largest buffalo and cow populations are located, with 16.019 million and 13.204 million herds, respectively. Punjab is also the major milk producing province with an annual production of 36.23 million litres, which corresponds to about 67% of total milk production in Pakistan [14]. Following data published by ISMEA (2023) [15], the regions selected in Italy were instead Lombardy, Piedmont, Emilia-Romagna, and Veneto, in which 80% of the total national cow population and milk production are concentrated. In the same regions, the highest milk consumption rate was established in 2023 [16].

### Survey design

**Fig. 2:**
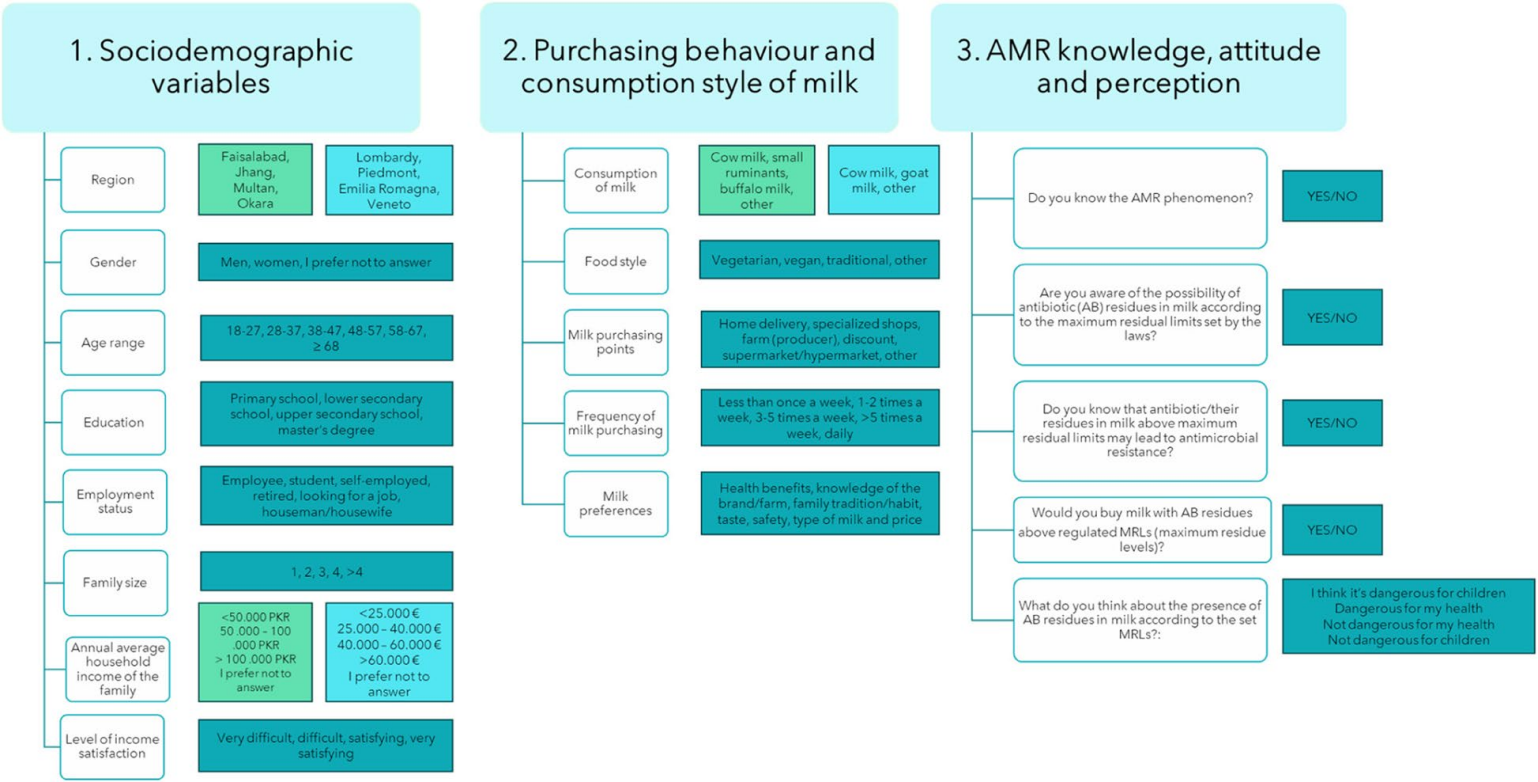
Survey questionnaires framework addressed to Pakistani and Italian consumers

Two consumer surveys were developed to collect data about Pakistani and Italian milk consumers in the two selected countries. Both surveys were developed into the same three sections, as shown in Fig. 2. The first section was designed to assess the socio-demographic profile of the respondents (i.e., gender, age, educational level, employment status, family size, annual average income, and level of income satisfaction). The second section explored the individuals’ food style and the purchasing behaviour and consumption habits of milk, using several double check-all-that-apply (CATA) and binary (Yes/No) questions. In addition, the preferences of consumers towards seven different characteristics of milk were measured using a 7-point Likert scale. The seven milk descriptors were selected by means of a thorough literature review, considering as a selection criterion the impact of each item in the decision-making process of milk consumers in both the selected countries: health benefits, knowledge of the brand/farm [17, 18], family tradition/habit, taste, safety, type of milk and price [19–22]. The last section consisted of five questions (both binary and categorical answers) related to consumer’s awareness about antibiotic residues in milk and AMR phenomenon. In general, the survey framework was formulated according to what reported in previous studies [23–26]. The structure of the surveys developed for the two countries was similar. However, some questions or possible answers were adapted to maintain the content validity across different social and economic-demographic contexts. In particular, the annual average income options were formulated based on the representative population data of the two countries [27, 28] (Fig. 2) and expressed in Pakistani Rupee (PKR) and in Euros (€) for the Pakistani and Italian sample, respectively. Moreover, due to the different milk consumption habits in the two countries, the functional unit of the surveys changed, being the Pakistani questions focused on both cow and buffalo milk, while the Italian questions were focused on cow milk only.

### Data collection

Two choice experiments were conducted in both countries through structured questionnaires submitted online to respondents from April to June 2022 using mailing lists, social networks, and WhatsApp. A non-probability sampling method was employed considering a convenience sample of milk consumers in the two countries [29, 30]. The inclusion criteria, required at the beginning of the questionnaire under penalty of survey exclusion, were the geographical affiliation and the respondent’s age (the legal age established in the countries). The questionnaires respected the ethical standards defined by the Declaration of Helsinki and were developed in the original language of each country (Urdu and Italian for Pakistan and Italy, respectively). The surveys were anonymous and did not include sensitive data. An informed consent was sought and obtained from each respondent prior to the participation in the survey. The questionnaires were approved by the Bioethical Committee of the University of Turin, Italy (approval protocol n. 0169932, 14/03/2022).

The sample size and the individual’s distribution in the two countries was determined based on practical constraints, including available resources and access to respondents. The sample was designed to capture a diverse cross-section of consumers in each country, focusing on obtaining a representative sample that reflects various demographic factors rather than proportional representation of the national populations. This sample size is consistent with similar studies in the field [31] and provides sufficient statistical power to detect meaningful differences and correlations in consumer attitudes.

### Statistical analysis

The software SPSS (version IBM SPSS Inc 28.0, Chicago, Illinois, USA) was used for all statistical analyses.

Frequency analysis was used to provide a descriptive summary of the socio-demographic characteristics of the Pakistani and Italian sample, as well as of the entire sample of respondents. Statistical difference between the two samples in terms of size and socio-demographic composition was tested using the Chi-square (χ^2^) test.

The Shapiro–Wilk test was used to determine whether the data followed a normal distribution.

The reliability of the scales of milk descriptors (seven items) was checked using the Cronbach's alpha test. A Principal Component Analysis (PCA) with Varimax rotation was performed starting from the variables of country, gender, family size, age, food style, frequency of milk purchase, and preferences towards milk attributes. PCA is a statistical data simplification technique that aims to reduce the number of variables that define a complex multivariate dataset to a smaller number of latent variables, while minimizing the loss of information and making the original data matrix easier to understand [32, 33]. This multivariate statistical methodology is commonly used in market research, especially in consumer studies, to assess people's tastes and opinions based on their purchasing behaviour and the characteristics of the products they use [20, 34–36]. The starting 12 variables were aggregated into a minor number of orthogonal principal components (PCs). The influence of a given variable in the component definition (expressed by the resulting loadings) was rejected for a value below 0.30, and it was considered to have a strong influence if it had a ratio above 0.7 [20]. KMO and Bartlett's tests were used to evaluate the reliability of the PCA, while the reliability of the new PCs were checked using the Cronbach's alpha test.

The PCA allowed to reduce the dimensionality of the dataset and to identify the PCs (consumption patterns) that explained most of the variance in the dataset regarding the attitudes and milk consumption styles/habits of the consumers involved by synthesizing the original information into a more manageable and interpretable form. The obtained PCs were the average of the individual weights (loadings, positive or negative) of the component per individual. A new matrix was obtained, consisting of a number of rows equal to the sample size and a number of columns equal to the number of PCs that contained the weight of the individual variable (loadings, positive or negative) per individual. To link the single new defined consumption pattern to each respondent, the loadings of each PC were used as dependent variables for a Cluster Analysis in order to trace consumption patterns back to individuals and identify distinct groups of consumers differing in terms of milk consumption styles and habits. This approach allowed to discriminate each individual in terms of milk consumption patterns.

The clusters were formed based on each individual's scores on the new identified PC. First, a two-step method was used to get a sense of how well the entire sample could be segmented using the Bayesian Information Criterion (BIC) and Akaike Information Criterion (AIC) indices. In terms of cohesion and silhouette separation, the test recommended segmentation with 4 clusters as the best option. Consequently, the K-Means clustering method was used to obtain the 4 homogeneous consumer groups in terms of socio-demographic characteristics, including country of origin, milk preferences, consumption patterns, and milk purchasing habits, which were different from each other. A one-way analysis of variance (ANOVA) was performed to evaluate the differences among the clusters. To define differences among the clusters, the groups were also analysed in terms of socio-demographic characteristics, and knowledge, attitude, and perception about AMR [37]. The Chi-square (χ^2^) test was used to evaluate clusters heterogeneity. This approach allowed us to group consumers according to similar patterns of attitudes, minimizing the loss of information resulting from the reduction of the original variables.

## Results

A total of 951 answers, including 401 from Pakistan and 550 from Italy, were collected.

### Consumers’ socio-demographic characteristics

Table 1 provides the socio-demographic profile of the involved consumers’ sample. The sample of milk consumers showed a significantly higher percentage of Italian than Pakistani respondents (*P* < 0.05). Most Pakistani individuals were from Faisalabad, while most Italian respondents were from Piedmont. Although significant differences have been detected between the two countries in terms of gender, age range and educational level (in all cases, *P* < 0.001), in both samples there were mostly men, aged between 18 and 27 years and with a high educational level (upper secondary school or master’s degree). Both samples were also prevalently characterized by a middle-high average income. Statistically significant differences in the responses between the two countries in terms of employment status and family size were also observed (in both cases, *P* < 0.001). Most Pakistani respondents were students, and families often had more than four members, while the Italian respondents were mostly employed with a family size on average consisting of three people. Both Pakistani and Italian respondents mostly defined their annual average income as satisfying.

**Table 1:** Socio-demographic features (%) of the Pakistani and Italian respondents, and of the entire consumers’ sample

Socio-demographic variables	Answers	Pakistan (*n* = 401)	Italy (*n* = 550)	Total (*n* = 951)	Χ^2^	Sig.^2^
		42.5	57.5	100	27.9	*
Region	Faisalabad	38.5	-	16.6	n.a	
	Jhang	18.2	-	7.7		
	Multan	26.6	-	10.8		
	Okara	16.7	-	6.9		
	Lombardy	-	30.3	17.2		
	Piedmont	-	39.2	23.6		
	Emilia Romagna	-	13.5	7.9		
	Veneto	-	17.0	9.2		
Gender	Man	57.8	51.8	51.9	21.0	***
	Woman	39.9	47.5	46.8		
	I prefer not to answer	2.3	0.7	1.3		
Age range	18–27	57.3	40.1	47.4	80.0	***
	28–37	22.4	13.8	17.4		
	38–47	10.6	13.0	12.0		
	48–57	5.8	16.5	12.0		
	58–67	3.3	10.8	7.6		
	≥ 68	0.6	5.8	3.6		
Education	Primary school	2.0	0.2	1.0	252.7	***
	Lower secondary school	3.0	8.2	6.0		
	Upper secondary school	28.5	42.6	36.6		
	Master’s degree	66.5	49.0	56.4		
Employment status	Employee	19.9	46.4	35.2	151.4	***
	Student	43.4	25.4	33.1		
	Self-employed	9.4	15.4	12.9		
	Retired	5.4	9.6	7.8		
	Looking for a job	12.0	1.9	6.1		
	Houseman/housewife	9.9	1.3	4.9		
Family size (number of family member)	1	0.5	22.7	13.1	937.0	***
	2	1.0	24.3	14.3		
	3	7.0	31.8	21.1		
	4	15.8	15.1	15.2		
	> 4	75.7	6.1	36.3		
Annual average household income of the family^a^	< 50.000 PKR	16.1	-	7.0	n.a	
	50.000 – 100.000 PKR	44.0	-	18.6		
	> 100 .000 PKR	39.9	-	16.8		
	< 25.000 €	-	16.7	9.8		
	25.000 – 40.000 €	-	32.3	18.6		
	40.000 – 60.000 €	-	18.7	10.7		
	> 60.000 €	-	8.2	4.8		
	I prefer not to answer	-	24.1	13.7		
Level of income satisfaction	Very difficult	5.8	1.9	3.5	24.0	***
	Difficult	19.6	15.0	17.0		
	Satisfying	64.0	77.0	71.5		
	Very satisfying	10.6	6.1	8.0		

^a^*PKR* Pakistani Rupee; €, Euros

^2^Significance level: ***P-value < 0.001; *P -value < 0.05. *n.a.* Not applicable

### Milk consumption patterns

Table 2 summarises the results of the PCA. A total of three PCs were extracted, accounting for 61.8% of the total explained variance.

**Table 2:** Results of the rotated component matrix

Variables	Principal Components		
	Milk quality attributes and tradition	Family-dependent	Eating style-dependent
Country		-0.919	
Family size		0.820	
Age (mean)			0.449
Food style			0.874
Frequency of milk purchasing		0.774	
Knowledge of the brand/farm	0.834		
Health benefits	0.774		
Price	0.669		
Family tradition/habit	0.672		
Taste	0.860		
Safety	0.876		
Type of milk		-0.646	
*Cronbach's Alpha*	*0.94*	*0.85*	
*Pearson’s correlation*			*r* = *0.799*

Varimax rotation; Kaiser–Meye–Olkin index = 0.822. Bartlett’s sphericity test: Chi-square = 4463.178; *P*-value = 0.000

The first component, renamed as "Milk quality attributes and tradition", accounted for the 31.4% of the total explained variance and yielded a consumption model in which milk quality characteristics are strongly considered in milk selection. This factor summarises a consumption pattern in which consumers choose milk primarily based on a combination of brand knowledge, health benefits, and safety, supported by secondary factors such as tradition, taste and price. In other words, these consumers are inclined to select products that they know and trust the brand, that they perceive as healthy and safe, and that satisfy their family and taste habits, while still considering price.

The second component, renamed as "Family-dependent" (22.0% of the total explained variance), identifies a consumption model depicted in function of family habits and composition. This consumption pattern reflects a condition where milk purchasing habits are strongly influenced by household size and the need for frequent purchases, while differences between countries and preferences for specific types of milk are less relevant.

The third and last component, renamed as "Eating style-dependent" (8.4% of the total explained variance), reflects a pattern of milk consumption influenced mainly by the consumers’ age and eating styles.

### Consumers’ profiles

The cluster analysis revealed four consumer groups, each including consumers sharing a similar attitude and understanding of milk (Table 3).

**Table 3:** Cluster definition: food consumption patterns derived by the PCA, and weight of each component in cluster definition, socio-demographics (%), purchasing behaviour and consumption style (%), and AMR knowledge, attitude and perception (%)

Variables	Clusters					
	Attentive to milk quality attributes (38.9%)	Loyal to milk (37.0%)	Undecided consumer (16.4%)	Milk is essential in my food pattern (7.7%)		Sig.^1^
***Food consumption patterns***					F	
Milk quality attributes and tradition	1.326	-1.304	-2.211	-0.564	590.669	***
Family-dependent	-1.450	1.683	-1.027	-0.865	661.252	***
Eating style-dependent	0.908	1.707	-0.777	5.350	542.098	***
***Socio-demographics (%)***						
					**χ**^**2**^	
*Country*						
Pakistan	0.63	98.36	33.33	74.19	625.539	**
Italy	99.37	1.64	66.67	25.81		
*Gender*						
Man	47.34	60.00	50.37	41.94	39.513	***
Woman	52.04	39.02	48.15	50.00		
I prefer not to answer	0.63	0.99	1.54	7.61		
*Age range*						
18–27	36.99	56.39	57.78	40.32	80.143	***
28–37	15.05	22.95	14.81	17.74		
38–47	12.23	11.80	8.15	14.52		
48–57	19.75	5.57	6.67	16.13		
58–67	10.66	2.30	8.15	8.06		
> 68	5.32	0.99	4.44	3.23		
*Education*						
Primary school	9.72	4.92	5.93	9.68	190.121	***
Lower secondary school	42.32	7.21	28.15	9.68		
Upper secondary school	0.00	21.31	5.19	17.74		
Master’s degree	47.96	65.57	60.74	61.29		
*Family size*						
1	24.45	0.00	12.59	8.06	405.043	***
2	22.26	0.66	18.52	11.29		
3	32.60	6.23	24.44	9.68		
4	15.05	16.39	15.56	17.74		
> 4	5.64	76.72	28.89	53.23		
*Level of income satisfaction*						
Very difficult	2.51	5.25	4.44	4.84	19.021	*
Difficult	14.73	20.00	12.59	22.58		
Satisfying	77.43	64.26	72.59	61.29		
Very satisfying	5.33	10.16	10.37	11.29		
***Purchasing behaviour and consumption style (%)***						
*Food style*						
Omnivore	96.31	99.35	92.86	0.00	22.456	n.s
Vegetarian	3.69	0.65	7.14	49.66		
Vegan	0.00	0.00	0.00	50.34		
*Frequency of milk purchase*						
Less than once a week	17.58	0.35	13.19	3.27	76.789	**
1–2 times a week	36.70	1.42	29.45	13.08		
From 3 to 5 times a week	18.81	5.54	5.52	8.41		
More than 5 times a week	5.50	3.97	7.36	7.48		
Daily	21.41	88.72	44.48	67.76		
*Milk consumption*						
Cow milk	90.03	65.78	90.57	78.95	32.643	*
Buffalo milk	2.11	32.62	6.60	15.79		
Small ruminant milk	7.85	1.60	2.83	5.26		
***AMR knowledge, attitude and perception (%)***						
*Do you know the AMR phenomenon?*						
Yes	99.37	37.38	88.89	67.74	50.581	***
No	0.63	62.30	11.11	29.03		
*Are you aware of the possibility of antibiotic (AB) residues in milk according to the maximum residual limits (MRL) set by the laws?*						
Yes	64.58	42.62	57.78	66.13	33.728	***
No	35.42	57.05	42.22	33.87		
*AB residues in milk over set MRLs can lead to AMR?*						
Yes	45.45	65.57	38.52	43.55	41.544	***
No	54.55	33.44	61.48	56.45		
*Would you buy milk with AB residues above regulated MRLs?*						
Yes	35.00	45.00	84.00	65.00	75.702	n.s
No	75.00	55.90	16.00	35.00		
*What do you think about the presence of AB residues in milk according to the set MRLs?*						***
I think it is dangerous for my health	19.12	54.75	20.74	41.94	50.581	***
I think it is more dangerous for children	42.01	35.08	45.93	29.03	41.544	***
I think it is not dangerous for my health	22.57	9.84	17.78	24.19	75.702	***
I don’t know	16.30	0.00	15.56	3.23	143.017	***

^1^ Significance level: *** *P*-value < 0.001; ** *P*-value < 0.01; * *P*-value < 0.05; n.s. = not significant

The first group was renamed as "Attentive to milk quality attributes" (38.9% of the total sample) because it positively depended on the components “Milk quality attributes and tradition” and “Eating style-dependent”, while it was negatively defined by the component “Family-dependent”. This cluster collected individuals interested in the quality characteristics of milk that probably represented a staple food in a defined dietary style. Most of these consumers were Italian, with a balanced representation of men and women. Educationally, the group was evenly split between those with higher education (48% with a master’s degree) and those with lower secondary education (42%). These individuals were predominantly young to middle-aged, with a satisfactory income level. They were mostly omnivores who purchase cow's milk several times a week or even daily. This group was the most informed about the AMR phenomenon and the least willing to purchase milk containing antibiotic residues above MRLs regulated by law. They were convinced of the negative effects of antibiotic residues in milk on children's health, although they were less concerned about their own health.

The second group, referred to as "Loyal to milk", comprised the 37.0% of the sample's overall population. This group was positively defined by both the PCs “Family-dependent” and “Eating style-dependent”, but negatively by the “Milk quality attributes and tradition” component.

This cluster was almost entirely composed of Pakistani consumers, particularly men, with a high level of education and belonging to large families. These individuals were young, omnivores, and had a satisfactory income level. Unlike the first group, their milk purchasing and consumption behaviour was strongly influenced by family needs and dietary habits rather than product quality. They consumed both cow and buffalo milk with high weekly frequency, showing a strong loyalty to milk consumption. This group was the least aware of the issues related to AMR and antibiotic residues in milk. Despite being convinced of the negative effects of antibiotic residues on human health, 45% of the respondents belonging to this group were still willing to consume milk with antibiotic residues above the regulated limits.

The third group, referred to as the "Undecided consumer" (16.4% of the total sample), was drawn negatively by all three obtained PCs and consisted of individuals who were probably not interested in milk characteristics and for whom neither type of eating habits or consumption patterns influenced the choice. They were mainly Italian consumers but also accounted for a good percentage of Pakistani consumers. They were especially young people, balanced in terms of both gender and family size. Their educational level was high with mainly satisfactory income level. They were mainly omnivores and were divided into two groups based on their milk purchasing frequency: high and low milk consumers. In terms of AMR awareness, most of them were informed about the phenomenon and the potential presence of antibiotic residues in milk. They also recognized that antibiotic residues in milk could contribute to antibiotic resistance in humans and believed that such residues are dangerous to both their health and children's health. However, about 16% of the respondents belonging to this group remained undecided about the effects of AMR.

The fourth group, referred to as "Milk is essential in my food pattern" (7.7% of the total sample), resulted in a substantial correlation with consumers’ eating habits. In fact, it was positively defined by the component “Eating style-dependent”, and negatively by the “Milk quality attributes and tradition” and “Family-dependent” components. This group, the least numerous one, was represented mainly by Pakistani consumers, balanced in terms of gender and age, belonging to large families and with a high education level. They often buy milk and consume especially cow milk. This group accounted for the higher percentage of vegan and vegetarian consumers. These consumers were aware about the AMR phenomenon and the possible presence of antibiotic residues in milk. In addition, a large proportion of them thought that antibiotic residues in milk are dangerous to human health.

## Discussion

This research provides a detailed overview of consumer awareness and concerns towards the problem of AMR in relation to the individuals’ socio-demographic characteristics and milk consumption patterns, including also the effect of the respondents’ geographical affiliation comparing a LMIC (Pakistan) and a HIC (Italy).

### Milk consumption pattern

The socio-demographic differences between milk consumers in Pakistan and Italy are in line with previous studies that highlighted how the cultural and socio-economic context influence eating habits. For example, an analysis conducted by Wardle et al. [38] showed that young adults tend to make food choices more based on convenience and cultural preferences than older generations. In our sample, most of the respondents were young adults, suggesting that their milk consumption habits may be influenced by both culture and affordability.

Overall, the PCA revealed a variety of complex and interconnected influences shaping milk consumption patterns in the two countries. The three obtained PCs provided significant insights into milk consumption dynamics in accordance with other studies that examined determinants of food choices [20, 39]. The first component, "Milk quality attributes and tradition", highlights a consumption pattern in which milk quality characteristics play a key role in product selection. This result is in line with published literature that emphasizes the importance of quality perception in food choice, especially for those commodities that are part of consumers' traditional diets [40]. The positive weight of the variables "health benefits", "price," "taste," and "safety" confirms the complexity of influences on milk choice [41, 42]. In addition, the positive correlation with the variables "family tradition" and "brand knowledge" indicates that family tradition and familiarity with the producer are determining aspects in milk choice. These results support the theory of "localness" in eating behavior, which suggests that consumers tend to prefer local and familiar food products [36, 43–45].

The second component, “Family-dependent”, shows how family size and habits influence milk purchasing habits. Studies by Chikweche et al. [46] and Ogundijo et al. [46, 47] showed that family size and supply needs significantly influence food purchasing decisions. Larger households buy milk more often and in larger quantities, regardless of their country of residence or available type of milk. Similar results were described in other studies, in which both the influence of the family context and the eating habits, especially as concerns food of daily consumption, influenced individuals’ food choices [43, 48].

The “Eating style-dependent” consumption pattern indicates that the age and eating style of consumers play a crucial role in milk consumption, highlighting how preferences and nutritional needs vary significantly among different age groups and dietary lifestyles, thus influencing the type and quantity of milk consumed. It is known that food preferences vary significantly with age and lifestyle, as reported by several authors [17, 29, 47, 49]. Our study confirms that young consumers often adopt strict eating styles, such as vegetarian or vegan. This result is in line with research that explored the effect of individual food preferences and dietary trends on food choice [50].

### Consumers’ heterogeneity and profiles: how do they perceive the AMR issue?

The use of the different PCs as variables in the cluster analysis yielded four distinct consumer profiles, each characterized by different socio-demographic features as well as by specific attitudes and behaviors towards milk and the AMR issue.

The **“**Attentive to milk quality attributes” cluster was predominantly represented by Italian consumers that valued the quality and tradition of milk as important aspects for milk choice. As reported by Camanzi et al. [51] and Krystallis [51, 52], quality-conscious consumers as the European ones are willing to pay more for products that they perceive as safe and healthy, in line with the European consumers that tend to value quality and food safety as the most important factors in their purchasing decisions. This group of consumers was the most informed about AMR and the least willing to buy milk containing antibiotic residues. This cluster could represent an ideal target group for premium products with quality certifications.

The “Loyal to milk” cluster mainly included Pakistani men from large families with high educational levels. This group was positively defined by the “Family-dependent” component, suggesting how the food pattern of this cluster was determined by the influence of family size and food habits. They consumed both cow and buffalo milk, and purchased milk frequently. This could be related to specific cultural and family traditions [40, 53, 54]. Most members of this group were young, heavy milk consumers who do not prioritize milk quality during purchase. This suggests that they were not the primary decision-makers but instead relied on the choices made by other family members, likely due to their young age. Consequently, the "Loyal to milk" group appeared indifferent to milk quality yet consumed large quantities of it as a habitual product within the family context. These consumers likely trusted the purchasing decisions made by others, making them less motivated to assess the quality and safety of the milk they consumed, which in turn rendered them more indecisive regarding the AMR issue. Furthermore, the limited attention of this group to milk quality characteristics may indicate a lack of awareness about the potential risks associated with consuming milk containing antibiotic residues. As a result, this cluster was more indecisive about AMR, despite recognizing its risks. Their perception of the risks associated with milk consumption is ambiguous. Although 45% of them were willing to consume milk with antibiotic residues above the legal limits, 62% claimed to be unaware of AMR. However, most of them believed that antibiotic residues above MRLs can lead to AMR and pose a risk to their health. Our data suggest that the "Loyal to milk" consumers had inadequate awareness of antibiotic residues and AMR, possibly influenced by their geographical background. As noted in previous research, inadequate surveillance systems and ineffective mitigation measures to reduce antibiotic use can decrease consumer awareness of AMR-related risks [55–57].

In parallel, their trust in a product containing antibiotic residues below pre-determined levels also suggests significant trust in institutions that protect public health by setting legal thresholds [58]. Education and awareness-raising can positively influence consumption habits; for instance, awareness of food safety issues is directly proportional to consumers' willingness to engage in responsible consumption behaviors [59]. Moreover, it is more evident in developing countries how low education about food safety leads to less responsible food choices by the consumer [60].

The "Undecided consumer" cluster did not show strong preferences influenced by any of the identified PCs. This group, comprising both Italian and Pakistani consumers, did not exhibit a strong preference for milk characteristics and seemed to base choices on family customs. The young age of this cluster suggests that they are more passive consumers rather than responsible buyers. Studies by Feindt and Poortvliet [61] and García-Maroto et al. [62] indicated that undecided consumers often lack sufficient information to make informed choices, suggesting that educational campaigns can positively influence their decisions. In the current study, the "Undecided consumer" group consisted of well-educated young individuals aware of the possible presence of antibiotic residues in milk. Nevertheless, they continued to consume large quantities of milk due to established habits. This behavior implies that theoretical knowledge about AMR does not always lead to changes in consumption habits, especially when these habits are deeply rooted. As suggested by Pentz et al. (2020) [61], low-risk perception may be due to the familiarity with the products, which justifies their continued consumption. Despite a certain level of awareness, the "Undecided consumer" group remains a target for information campaigns aimed at improving understanding of AMR-associated risks.

The difference between the "Undecided consumer" and "Loyal to milk" groups is significant: the latter is characterized by simultaneous disinterest in both quality and family-influenced eating habits. The "Undecided consumer" group, frequently mentioned in consumer studies literature, often consists of demotivated individuals who choose a diet plan without deep-rooted motivations and are ready to change styles and habits. They may consume milk simply because they enjoy it. The key difference with the "Loyal to milk" group, besides the definition in the weighting of individual PCs, lies in their level of knowledge about AMR: the "Undecided consumer" group appeared confused, knowing about AMR but not perceiving its danger.

The “Milk is essential in my food pattern” cluster included Pakistani consumers, especially vegans and vegetarians, highly educated, and who frequently purchase milk probably for their family, in accordance with their food style. Like the “Loyal to milk” individuals, consumers in this cluster also considered milk essential, but their consumption is more closely tied to individual dietary habits and lifestyle choices, such as health consciousness or dietary needs. Unlike the "Loyal to milk" group, this cluster places greater emphasis on the perceived health benefits of milk and its role in their overall food pattern, which may influence their preference for specific types of milk or brands. These differences underscore the nuanced motivations behind milk consumption among different consumer segments. They have high AMR awareness, but a more relaxed attitude towards the potential presence of antibiotic residues in milk. Despite their high awareness of AMR, the consumers belonging to this cluster believe that antibiotic residues are not hazardous to human health. This group could benefit from targeted health campaigns addressing the risks associated with the presence of antibiotic residues in milk.

Both the “Loyal to milk” and “Milk is essential in my food pattern” clusters were mainly represented by Pakistani consumers and were less concerned about the presence of antibiotic residues in milk than their Italian counterparts. The result could imply limited awareness of the potential risks associated with the consumption of contaminated milk and/or spread of AMR linked to the geographical affiliation. The differences in food safety and consumer regulatory regimes between Italy and Pakistan are significant and likely contribute to the observed variations in consumers’ attitudes and behaviors. The stringent regulations and cultural practices around food consumption in Italy may foster a more health-conscious and regulated approach to milk consumption, while the diverse and less regulated Pakistani context may lead to varied consumption patterns influenced by cultural and economic factors. At the same time, worthy of note, although there are international rules for MRLs in milk, Pakistani dairy farmers often do not follow these rules [62]. Our results reflect those by Farhan et al. [62] who investigated dairy farmers’ awareness of antibiotic use in livestock farming systems in Pakistan. In this country, self-medication and misuse of prescribed antimicrobials are quite common. According to Ramesh and Tripathi [63], Indian milk consumers were similar to Pakistani ones as they were also unaware of the potential presence of antibiotic residues in milk, and they also showed significantly low concern about the potential health-associated risks. Studies showing low awareness of AMR through the food chain have also been conducted in various other LMICs, including Ethiopia [64], Sudan [65], Jordan [66], and Chile [67]. In contrast to the above-mentioned studies, in the current trial the Pakistani sample had a higher percentage of young respondents who were educated and knowledgeable about AMR. This finding suggests that education is a key factor in increasing awareness of AMR in LMICs, as also observed in India [63], Thailand [68], Chile [67] and Jordan [66].

## Conclusions

Our data demonstrate that both the geographical affiliation and the age of consumers are crucial and impacting drivers on AMR awareness and concern. Namely, statistically significant differences on sample distribution have been observed between Pakistani and Italian consumers, the first ones being more loyal to milk and the latter more aware about milk quality. The four clusters identified in our analysis are clearly differentiated by socio-demographic characteristics and attitudes towards milk and AMR. The first group, "Attentive to milk quality attributes", is composed primarily of Italian consumers, evenly split by gender and age, with a strong focus on milk quality and high awareness of AMR. The second group, "Loyal to milk", consists mostly of young Pakistani men, influenced by family needs and dietary habits, and shows less awareness of AMR compared to the first group. The third group, "Undecided consumer", includes both Italian and Pakistani consumers who show less interest in milk characteristics and are somewhat undecided about the effects of AMR, although being generally well-informed. Finally, the fourth group, "Milk is essential in my food pattern", is mainly composed of Pakistani consumers, often vegetarians or vegans, who view milk as an essential part of their diet and have moderate awareness of AMR. Each cluster represented a different nuance in attitudes toward milk and, despite some similarities, our analyses show that the clusters are distinct based on key components, such as sociodemographic features, awareness about AMR and purchase preferences. In particular, our results provide innovative insights into milk consumption patterns and consumer profiles in the two studied countries, highlighting socio-demographic and cultural differences that emerged having significant implications for marketing strategies and public health policies. Our study highlights significant differences in the KAP of milk consumers in Italy and Pakistan. Italian consumers exhibit high levels of knowledge and a generally positive attitude towards milk, though health concerns are influencing a shift in consumption practices. In Pakistan, while attitudes remain positive, knowledge gaps and risky consumption practices persist, especially in rural regions. These findings underscore the need for targeted interventions in both countries to address specific KAP-related challenges.

Our findings represent an interesting starting point for future surveys devoted to deepening how the cultural, educational, and dairy product accessibility can influence food preferences. Indeed, tailored communication and marketing strategies could give, in both cultural backgrounds, an effective improvement on public health promotional campaigns aimed at promoting healthier and more sustainable eating behaviors. Understanding these distinctions can inform targeted marketing strategies and public health interventions in both countries. In fact, based on the findings of our study, we recommend reinforcing public health messages in Italy to support balanced milk consumption and addressing emerging health concerns. In Pakistan, there is a critical need for educational campaigns to improve milk safety knowledge, particularly in rural areas, and to promote safer consumption practices through increased access to pasteurized milk. In Pakistan, education is a key factor in promoting awareness, whereas in Italy, external forces, such as government policies and public and private organizations, play a more significant role. Survey findings may help to identify areas of intervention and to boost new policies. As concerns LMICs, there is the need to strengthen consumers’ education to build awareness of food safety. On the other hand, milk consumers' expectations and concerns can influence the enactment of new regulations consistent with their demands, and consequently can positively affect farming practices and milk safety.

Thus, addressing the global challenge of AMR requires a concerted effort to educate and raise awareness among consumers, especially in LMICs. By fostering a better understanding of AMR, we can promote safer consumption practices and enhance the effectiveness of public health interventions, thereby contributing to a healthier global community and an effective One Health approach.

### Limitations of the study

The goal of the current study was to compare the socio-demographic features and habits of Pakistani and Italian milk consumers, understanding how the above-mentioned factors affect their knowledge, attitude and perception of the AMR issue. Even if the cluster analysis provides an in-depth perspective of milk consumption models in two different cultural contexts, the study has potential limitations. This study provides valuable insights into consumer attitudes in Italy and Pakistan, although the findings may not be generalizable to all developed or developing countries. Future research could expand the geographical context to include a wider range of countries to enhance the global applicability of the obtained results.

It is well known that the use of an online survey implies self-selection and coverage bias as questionnaires are generally completed by people interested in the topic and who have Internet access. As representativeness of the online data is debatable, caution should be used in generalizing the results to a broader population. In addition, while the sample size of 951 respondents provides valuable insights into consumers’ attitudes in Italy and Pakistan, it is important to acknowledge that the findings may not fully represent the entire national populations of these countries. Future research could explore larger and more proportionally representative samples to validate and extend these findings.

Finally, although the age distribution of the respondents is in line with the composition of the population in the two countries, the educational level of the Pakistani sub-sample does not reflect (being higher) the actual educational level of the overall Pakistani population.

## Supplementary Information

Supplementary Material 1

Supplementary Material 2

## Data Availability

No datasets were generated or analysed during the current study.
