# Supplementary material for: Milk consumers in Pakistan and Italy: a comparative study on the effects of geographical affiliation, socio-demographic characteristics and consumption patterns on knowledge, attitude and perception of antimicrobial resistance
Source: BMC Public Health. 2024 Dec 18;24:3463. doi: 10.1186/s12889-024-21002-w (PMC11657578; doi:10.1186/s12889-024-21002-w)
Supplement: Supplementary file 1 — Supplementary Material 1 [file 12889_2024_21002_MOESM1_ESM.pdf]

Dear Sir or Madam,

the questionnaire that is proposed to you is part of a survey developed by the Department of Agricultural, Forest and Food Sciences of the University of Turin (Italy). The objective of this research is to explore the milk consumer's awareness regarding the use of antimicrobials in dairy farms. In addition, the survey investigates the habits of choice and consumption, as well as the preferences, of milk consumers. To this end, the questionnaire is organized into 2 main sections:

- the first section is focused on the socio-demographic characteristics of the respondent and its cow/buffalo milk purchasing and consumption habits.
- the second section relates to the level of knowledge of the use of antimicrobials for the management of dairy cattle/buffaloes.

The collected data are totally anonymous and will not be used for profit purposes. The collected data will be computerized and processed through the support of statistical packages by the Researchers involved in this study: Prof. Manuela Renna (Associate Professor of Animal Nutrition; scientific manager of the project), Dr. Valentina Maria Merlino (Research fellow) and Dr. Talal Hassan (Student of the Master' Degree Course of Animal Science).

The completion of the questionnaire will take you 3-4 minutes at most.

Feel free to answer the questionnaire or refuse to fill it out at any time you wish.

In the next section you will have to choose whether or not to consent to fill out the questionnaire.

If you click "I consent," you can proceed with the completion. Otherwise, you can decide to abandon the completion by choosing the "I do not consent" option.

- I consent

- I do not consent

Thank you.

a. General information

1. Where do you live? (indicate the region)

a) Lombardy

b) Piedmont

c) Emilia Romagna

d) Veneto

2. You are

a) Man b) Woman c) I prefer not to answer

3. Age: ______________________________________

4. Family size:

a) 1 b) 2 c) 3 d) 4 e) more than 4

5. Age of Children

1. Son/Daughter _______ 2. Son/Daughter _________ 3. Son/Daughter________

6. Employment status

o Student	o Employee	o Self-employed	o Retired	o Looking for a job	o Housewife or houseman
Other then please specify____					

7. Education Level/Qualification:

o Primary School	o Lower Secondary School	o Upper Secondary School	o Master Degree

8. Indicate the annual average family income:
☐ less than 25.000 € ☐ between 25.000 and 40.000 € ☐ between 40.000 and 60.000 €
☐ more than 60.000 € ☐ I prefer not to answer

9. How can you define your income?

Difficult	Very difficult	Satisfying	Very Satisfying

b. Milk purchasing and consumption habits

10. Do you consume milk?

a) YES b) NO

11. What type of milk do you consume?

o Cow milk	o Goat milk	Other? Specify ___________________

12. What is your food style?

o Vegetarian	o Vegan	o Traditional
o Other. Specify________________________________		

13. Where do you buy cow/buffalo milk from? (More than one answer is possible)

o Home delivery (Gawala)/	o Milk shop	o Farm (Producer)/
o Supermarket	o Other? Specify__________	

14. How many times do you buy milk?

o Less than once a week	o 1-2 times a week	o From 3 to 5 times a week	o More than 5 times a week	o Daily

15. Considering a scale from 0 (not at all important) to 6 (very important), indicate the level of importance that you consider for each of the following factors during the milk choice in the purchasing-process:

Factors	0	1	2	3	4	5	6
Producer reliability							
Knowledge of the farmer							
Health benefits							
Have no alternative							
Price							
Family tradition							
Approval of family head							
Taste							
Safety							

c. Antibiotics residues in milk

16. Are you aware of the possibility of antibiotic (AB) residues in milk according to the maximum residual limits set by the laws?

a) Yes b) No

17) Do you know that antibiotic/their residues in milk above maximum residual limits may lead to antimicrobial resistance?

a) Yes b) No

18. What do you think about the presence of antibiotic residues in milk:

a. I think it is dangerous for my health
b. I think it is more dangerous for children.
c. I think it is not dangerous for my health
d. I don’t’ know

19. Would you buy milk with AB residues above regulated MRLs (maximum residue levels)?

a) Yes b) No c) I do not know

20. Do you know the AMR phenomenon?

a) Yes b) No

If not, we explain it to you. Antimicrobial resistance refers to the ability of microorganisms to withstand antimicrobial treatments. The overuse or misuse of antibiotics has been linked to the emergence and spread of microorganisms which are resistant to them, rendering treatment ineffective and posing a serious risk to public health.

Thank you!

The questionnaire is finished.

Thank you for giving a few minutes of your time to this research.

Best regards.
